# Selection Criteria for Cochlear Implantation in the United Kingdom and Flanders: Toward a Less Restrictive Standard

**DOI:** 10.1097/AUD.0000000000000901

**Published:** 2020-06-22

**Authors:** Tirza F. K. van der Straaten, Jeroen J. Briaire, Deborah Vickers, Peter Paul B. M. Boermans, Johan H. M. Frijns

**Affiliations:** 1 Department of Otorhinolaryngology and Head & Neck Surgery, Leiden University Medical Center, Leiden, The Netherlands; 2 Department of Clinical Neurosciences, University of Cambridge, Cambridge, United Kingdom; 3 Leiden Institute for Brain and Cognition, Leiden University, Leiden, The Netherlands.

**Keywords:** Adults, Cochlear implants, Deafness, Hearing loss, Post-lingual, Selection criteria

## Abstract

The impact of the newly introduced cochlear implantation criteria of the United Kingdom and Flanders (Dutch speaking part of Belgium) was examined in the patient population of a tertiary referral center in the Netherlands. We compared the patients who would be included/excluded under the new versus old criteria in relation to the actual improvement in speech understanding after implantation in our center. We also performed a sensitivity analysis to examine the effectiveness of the different preoperative assessment approaches used in the United Kingdom and Flanders.

**Design:**

The selection criteria were based on preoperative pure-tone audiometry at 0.5, 1, 2, and 4 kHz and a speech perception test (SPT) with and without best-aided hearing aids. Postoperatively, the same SPT was conducted to assess the benefit in speech understanding.

**Results:**

The newly introduced criteria in Flanders and the United Kingdom were less restrictive, resulting in greater percentages of patients implanted with CI (increase of 30%), and sensitivity increase of 31%. The preoperative best-aided SPT, used by both countries, had the highest diagnostic ability to indicate a postoperative improvement of speech understanding. We observed that patient selection was previously dominated by the pure-tone audiometry criteria in both countries, whereas speech understanding became more important in their new criteria. Among patients excluded by the new criteria, seven of eight (the United Kingdom and Flanders) did exhibit improved postoperative speech understanding.

**Conclusions:**

The new selection criteria of the United Kingdom and Flanders led to increased numbers of postlingually deafened adults benefitting from CI. The new British and Flemish criteria depended on the best-aided SPT with the highest diagnostic ability. Notably, the new criteria still led to the rejection of candidates who would be expected to gain considerably in speech understanding after implantation.

## INTRODUCTION

In postlingual adults with severe to profound hearing loss (HL), the general goal of a cochlear implant (CI) is to improve speech understanding. When setting selection criteria, the aims are to ensure that CIs are provided to candidates who are likely to benefit in speech understanding, while avoiding unnecessary costs and medical intervention for patients for whom acoustic hearing aids (HAs) are sufficient. Over recent years, technological developments and changes in surgical techniques have enabled the preservation of residual hearing and improved postoperative speech outcomes (Blamey et al. 2013; Snel-Bongers et al. 2018). However, improvement of speech understanding remains challenging when CI candidates have residual hearing and exhibit relatively high preoperative scores. For these borderline candidates, defining selection criteria for CI is a difficult process and is often based on expert opinions.

CI selection criteria show substantial variation at the international level (Friedland et al. 2003; Cullen et al. 2004; Dowell et al. 2004; Verhaegen et al. 2008; De Raeve & Wouters 2013; Hughes et al. 2014; Leigh et al. 2016; Vickers et al. 2016; Gubbels et al. 2017; Maeda et al. 2018; McRackan et al. 2018; Snel-Bongers et al. 2018; Huinck et al. 2019). Such candidacy criteria are commonly based on the anticipated postimplant speech outcomes, with cutoff values for preoperative criteria defined using the lowest 10th to 25th percentiles (p10–p25) (Dowell et al. 2004; Verhaegen et al. 2008; Snel-Bongers et al. 2018) or the proportions of patients with and without postoperative improvement in speech understanding (e.g., 1/4 patients may have no benefit postimplantation). However, there is also still a tendency to use conservative CI selection criteria to preserve a benefit in speech understanding postimplantation. For example, a conservative selection criterion would be an average of 85 dB or higher at 0.5, 1, and 2 kHz and a maximum phoneme score of 30% with HAs (Huinck et al. 2019).

When aiming to improve speech understanding after CI, the preoperative level of speech understanding is the most valuable indicative measure to use for CI selection criteria (T. F. K. van der Straaten et al., Reference Note 1). However, the types of preoperative audiometric and speech measures used to assess CI candidacy vary widely. For example, the United States applies a broad spectrum of preoperative measures (e.g., sentence or word tests) and selection criteria across the country (Friedland et al. 2003; Cullen et al. 2004; Hughes et al. 2014; Gubbels et al. 2017; Holder et al. 2018). Developing countries tend to exclusively use pure-tone audiometry (PTA) due to the accessibility. In general, a patient’s degree of HL and benefit from acoustic HAs is frequently determined via a combination of PTA and speech perception tests (SPTs) (Vickers et al. 2016).

Until recently, the United Kingdom and Flanders (the Dutch speaking part of Belgium) used relatively conservative criteria compared with the Netherlands, Germany, and Australia (Vickers et al. 2016), but both have recently developed new criteria (Table 1) (National Institute for Health and Clinical Excellence 2009; De Raeve & Wouters 2013; National Institute for Health and Clinical Excellence 2019). The new British criteria were driven by a panel of experts who reviewed the available evidence and provided recommendations. To determine the best SPT, a National Service Evaluation was conducted to collect SPT and PTA scores from adults with CIs, both preoperatively and up to 1 year postoperatively (Vickers et al. 2016). In addition, research comparing outcomes of children with CIs versus HAs provided evidence for shifting thresholds to an 80-dB level of HL (Lovett et al. 2015); however, this was based on children who were implanted under the prior conservative criteria (90 dB HL at 2 and 4 kHz). The National Institute for Health and Clinical Excellence has stated that the newly introduced criteria will help to better identify CI candidates. They predicted a 70% increase of patients under the updated recommendation. The new Flemish criteria were also recently implemented, to replace their outdated and conservative previous criteria (De Raeve & Wouters 2013).

**TABLE 1.** Selection Criteria for Cochlear Implant Candidacy by the United Kingdom and Flanders

	Old Criteria	New Criteria
United Kingdom	>90 dB at 2 and 4 kHz and <50% sentence score (2009)	≥80 dB at ≥2 frequencies (0.5, 1, 2, 3, and 4 kHz) and <50% phoneme score (2019)
Flanders	Average of >85 dB at 0.5, 1, and 2 kHz and <30% phoneme score (2013)	Average of >70 dB at 0.5, 1, 2, and 4 kHz and <50% phoneme score (2019)

In the present retrospective study, we aimed to evaluate the effects of using new selection criteria for CI patient selection in the United Kingdom (January 2019) and Flanders (August 2019) and to compare the postoperative gains in speech understanding among CI candidates, based on the outcomes of a large group of patients (n = 552) implanted under relatively lenient criteria at the Leiden University Medical Center (LUMC), a tertiary referral center in the Netherlands. Each country uses a different combination of PTA and SPT to evaluate the degree of HL and the benefit from acoustic HAs. It is of interest to determine the extent to which both selection criteria contribute to identifying the candidates who will benefit most from implantation. We expected to find that higher percentages of patients, who exhibited improved speech understanding postoperatively, were accepted under the new criteria compared with the old criteria. In addition, we examined the diagnostic values of the different preoperative measurement approaches used by the United Kingdom and Flanders, using sensitivity and specificity analyses.

## MATERIALS AND METHODS

### Procedure

In this retrospective study, we reviewed all adults with postlingual HL who were implanted with CI at the LUMC (ethical approval was obtained through the Medical Ethics Committee of the LUMC). Postlingual HL was defined as the onset of moderate to profound HL (>40 dB) after 4 years of age. In total, we reviewed the records of 566 patients with bilateral postlingual HL, who had CI implanted between 2000 and 2017, and who were 18 years of age or older at the time of implantation. The second side of patients with sequential bilateral implantation were excluded from analysis (n = 4). All patients had to have a postoperative follow-up of at least 1 year. Fourteen patients were consequently excluded, of whom five were explanted within 1 year (because of partial luxation or migration of the electrode, implant failure, wound infection, or removal of vestibular schwannoma), seven died (due to causes unrelated to implantation) during the first year, and two (one of them a marginal performer) were lost to follow-up after 3 months, precluding conclusions about their final outcomes. After exclusions, our analysis included 552 postlingual patients. Table 2 presents descriptive statistics of this study population.

**TABLE 2.** Descriptive Statistics of the Study Population (n = 552)

Age at implantation, yr, mean (SD)	60.6 (14.6)
Duration of hearing loss, yr, mean (SD)	33.9 (18.2)
Duration of severe bilateral hearing loss, yr, mean (SD)	19.4 (17.5)
Sex, n (%)	
Male	241 (43.7)
Implantation side, n (%)	
Right	295 (53.4)
Left	248 (44.9)
Bilateral	9 (1.6)
Manufacturer and Electrode type, n (%)	
Advanced Bionics (Los Angeles, CA)	460 (83.3)
Clarion II implant with HiFocus1 electrode	49
HiRes 90K implant with HiFocus1J electrode	233
HiRes 90K implant with HiFocusMS electrode	178
Cochlear (Sydney, Australia)	49 (8.9)
Nucleus Freedom with Contour Advance electrode	24
Nucleus Freedom with Hybrid-L24 electrode	25
MED-EL (Innsbruck, Austria)	43 (7.8)
Concerto implant with Medium electrode	36
Concerto implant with Flex electrode	7
Cause of deafness, n (%)	
Hearing loss with unknown cause	193 (34.3)
Genetic hearing loss	185 (32.9)
Infectious	84 (14.9)
Sudden deafness	48 (8.5)
Middle ear problems	31 (5.5)
Other	21 (3.7)

### Preoperative Measures

PTA was performed using frequencies of 0.5, 1, 2, and 4 kHz to calculate the preoperative degree of HL. In addition, speech understanding scores were conducted using the standard Dutch SPT of the Dutch Society of Audiology, which comprises phonetically balanced monosyllabic consonant-vowel-consonant words (Bosman & Smoorenburg 1995). First, we determined the maximum unaided phoneme score (over headphones). Next, we determined the phoneme and word score using best-fitted HAs in the free field at 65 dB and 75 dB SPL or with a +5 dB signal-to-noise ratio. The standard testing procedure comprised four lists, containing 11 words per condition (a total of 44 words and 132 phonemes). In the free field, words were presented through a loudspeaker set 1 m in front of the patient. If a patient achieved a phoneme score above 50% in a quiet setting, a speech-in-noise test was conducted in speech-shaped noise at a +5 dB signal-to-noise ratio.

### Selection Criteria of the LUMC

At the start of the CI program in 2000, the auditory candidacy criteria included a pure-tone average HL of >90 dB at 0.5, 1, 2, and 4 kHz in the better ear and best-aided (with one or two HAs) speech understanding of ≤30% phonemes correct in a quiet setting, corresponding to a 10% word score. These criteria changed over time. Since 2012, the selection criteria have been based only on SPT, with patients having phoneme scores in a quiet setting of ≤60%, and from 2016 onward of ≤80% (≤60% word scores) considered as CI candidates (Snel-Bongers et al. 2018). An additional criterion for candidates with >50% phoneme score in quiet was that they should have a phoneme score <50% in a +5 dB signal-to-noise ratio. The worst-performing ear was often implanted to preserve the best-performing ear for HA usage.

### Postoperative Outcome Measure

During the first 3 months following implantation, patients received intensive hearing rehabilitation from professional speech therapists. Postoperative follow-up occurred at 1 and 2 weeks; 1, 3, and 6 months; and 1, 2, and 3 years after surgery. Postoperative and follow-up examinations included testing of only the implanted ear, with an unaided or plugged contralateral ear, to examine the actual CI progress. Improvement in speech understanding was analyzed by subtracting the best-aided preoperative phoneme and word score at 65 dB and 75 dB SPL from the postoperative phoneme and word scores with CI at the same presentation level.

### Statistical Analysis

We modified the preoperative PTA and SPT to be comparable to the selection criteria of the United Kingdom and Flanders (National Institute for Health and Clinical Excellence 2009; De Raeve & Wouters 2013; National Institute for Health and Clinical Excellence 2019). Different PTA frequencies were utilized, and we calculated the average of SPT at 65 and 75 dB to approximate the SPT at 70 dB used in these two countries. Using the conversion formula of Vickers et al. (2013), we converted the 50% score on the Bamford-Kowal-Bench sentence test from the old British criterion to a 30% phoneme score on the Arthur Boothroyd word test (Vickers et al. 2013). This test provides a phoneme score that is highly comparable to Dutch phoneme scores. Flanders uses the same Dutch CVC word list for evaluating speech understanding, ensuring direct comparison with our data (Bosman & Smoorenburg 1995).

We used the old and new CI selection criteria of the United Kingdom and Flanders to separate the study sample into different groups: excluded or included according to the old and new criteria (Table 1). Descriptive analyses were performed, and a graphical scatter plot was generated with the included and excluded patients plotted against the improvement of speech understanding after CI. We evaluated the performance of preoperative measurements for predicting benefit using receiver operator characteristic (ROC) curve analysis. An ROC curve is a graphical plot that illustrates the diagnostic ability of a measurement with a binary outcome (improvement of speech understanding ≥0% or no improvement after CI), as its discrimination threshold is varied (Lasko et al. 2005; Fawcett 2006). Data analyses were performed using the IBM SPSS Statistics 26.0 software package.

### Missing Data

Missing data were analyzed with Little’s missing completely at random test (Little 1988). The result was significant (*p* < .001) meaning that the missing data were either missing at random or missing not at random. Missing at random would indicate that the underlying reason for missing data was related to known patient characteristics, which was the case in our study. Most patients with missing data were either good or poor performers, such that their yearly appointments were deemed unnecessary. We were missing 1-year postoperative SPT results from a quiet setting from 136 patients and from a setting with noise from 221 patients. Incomplete cases are automatically excluded from standard analyses, such as ROC curves (Netten et al. 2017). However, excluding these patients might bias the findings and potentially lower the power of the results. Thus, we applied a multiple imputation technique to impute the missing data based on known patient characteristics (gender, age at implantation, implantation side, deafness duration, deafness cause, preoperative PTA, preoperative SPT, and postoperative SPT at other follow-up evaluations) (van Buuren 2012). Ten datasets with imputations were produced. All ROC curves were generated using both the imputed and original datasets, revealing no differences in outcomes. The original dataset, including 416 patients with CI and postoperative scores at 1 year, was used for descriptive analyses and scatter plots.

## RESULTS

### Postoperative Speech Understanding Scores

**Fig. 1.**
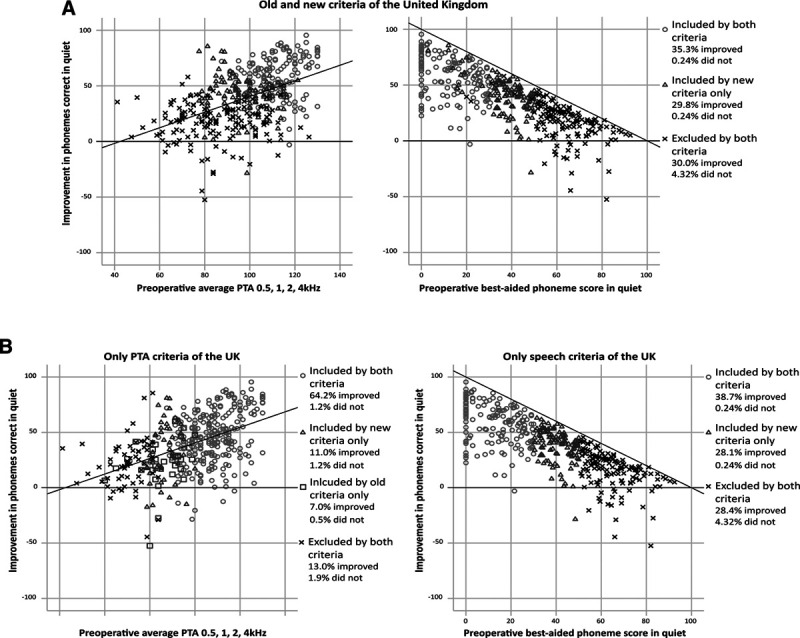
Numbers of patients included or excluded by the selection criteria in the United Kingdom and Flanders (raw data, n = 416). A and C shows the combination of preoperative pure-tone audiometry (PTA) and speech perception test as selection criteria. B and D illustrates each individual preoperative measure as the selection criterion.

At 1 year postoperatively, 396 patients (95.2%) exhibited improvement and 20 patients (4.8%) did not show improvement of their speech understanding in a quiet setting (70 dB SPL) with their CI. The postoperative improvement in speech understanding exhibited a ceiling effect, indicating that abundant improvement was not possible in patients with high preoperative best-aided phoneme scores (reference line in Fig. 1). Among the 20 patients without improved speech understanding in a quiet setting, two patients exhibited improved speech understanding in the setting with noise (difference in phoneme score of 5% and 18% at the +5 dB signal-to-noise ratio) and 15 patients exhibited improved speech understanding on the side of implantation (mean improvement from maximum phoneme score: 18%; SD, 28%). Three patients (0.7% of the total population) exhibited no improvement of speech understanding at any level after implantation (difference in phoneme score at 70 dB: −1%, −14%, and −16%).

### Included or Excluded by the Selection Criteria of the United Kingdom and Flanders

Figure 1 shows the range of preoperative degree of HL and best-aided phoneme scores plotted against the postoperative improvement of speech understanding for each criteria.

The new selection criteria of the United Kingdom led to the inclusion of 30% more patients, of whom 0.2% did not exhibit postoperative improvement of speech understanding (Fig. 1A). This new group exhibited a 41% improvement of speech understanding, in contrast with the 59% improvement within the group accepted based on the old criteria (Table 3). Among all analyzed patients, 34.4% were excluded by both the old and new selection criteria. In this excluded group, one of eight patients (4.3%) did not exhibit postoperative improvement, and this group improved their speech understanding by an average of 17%. Overall, our findings indicated that the new British criteria result in the selection of patients who will have a postoperative improvement, excluding two patients who showed a >50% improvement postoperatively.

**TABLE 3.** Postoperative Improvement of Speech Understanding Among the Candidates Included or Excluded by the Selection Criteria of the United Kingdom and Flanders (Raw Data, n = 416)

	United Kingdom			Flanders		
	Included by Both Criteria	Additionally Included by New Criteria	Excluded by Both Criteria	Included by Both Criteria	Additionally Included by New Criteria	Excluded by Both Criteria
n (%)	148 (35.6)	125 (30.0)	143 (34.4)	151 (36.34)	126 (30.24)	139 (33.42)
Mean improvement (SD)	59.1% (18.8%)	41.2% (16.6%)	16.7% (17.3%)	59.5% (18.6%)	40.4% (15.9%)	15.8% (16.7%)
Range	−3% to 96%	−29% to 86%	−53% to 58%	−3% to 96%	−28% to 81%	−52% to 46%

The new selection criteria of Flanders led to the inclusion of 30.2% more patients, of whom 0.2% did not exhibit postoperative improvement of speech understanding (Fig. 1C). This group of newly included candidates exhibited a 40% improvement of speech understanding on average, as opposed to an improvement of 60% among patients who would be included by both the old and new criteria (Table 3). Among all analyzed patients, 33.4% would be excluded by both the old and new selection criteria of Flanders. Within this excluded group, one of eight patients (4.3%) exhibited no postoperative improvement, and this group exhibited a 16% postoperative increase of speech understanding. On the other hand, the new criteria of Flanders included all patients who showed >50% improvement of speech understanding postoperatively.

Next, the selection criteria were separated by the PTA and speech prerequisites (Fig. 1B, D). The new criteria of the United Kingdom relied more on the speech criterion than on the PTA criterion, because the amount of patients excluded almost corresponded when following both PTA and speech criteria versus following only the speech criteria. There were five instead of 12 additional patients excluded based on the PTA prerequisites of the new and old criteria, respectively, on top of the patients excluded based on the speech criteria (Fig. 1A). Moreover, following only the PTA prerequisites of the United Kingdom resulted in a considerable amount of patients (65.4%) included by both the old and new criteria (Fig. 1B). Notably, a small group of patients (7.5%) were excluded by the new PTA criterion but were included by the old PTA criterion.

The new criteria of Flanders were also predominantly based on the speech criterion, because the amount of patients excluded nearly resembled the amount when following both PTA and speech criteria or the speech criteria alone. Two instead of 11 additional patients were excluded based on the PTA conditions when following the new instead of the old criteria on top of the patients excluded based on the speech criteria (Fig. 1C). Following only the PTA condition of Flanders resulted in a substantial amount of patients (70.4%) included by both the old and new criteria (Fig. 1D).

### Performance of Preoperative Measures

The change of British criteria led to a sensitivity increase from 37.1% to 68.4% (respectively, 147 and 271 patients who were included and improved postoperatively) and a specificity decrease from 95% to 90% (respectively, one and two patient(s) who were excluded and did not improve postoperatively). The change of Flemish criteria had an identical decrease of specificity and a similar increase of sensitivity from 37.9% to 69.4% (respectively, 150 with the old criteria and 275 patients with the new criteria who were included and improved postoperatively).

**Fig. 2.**
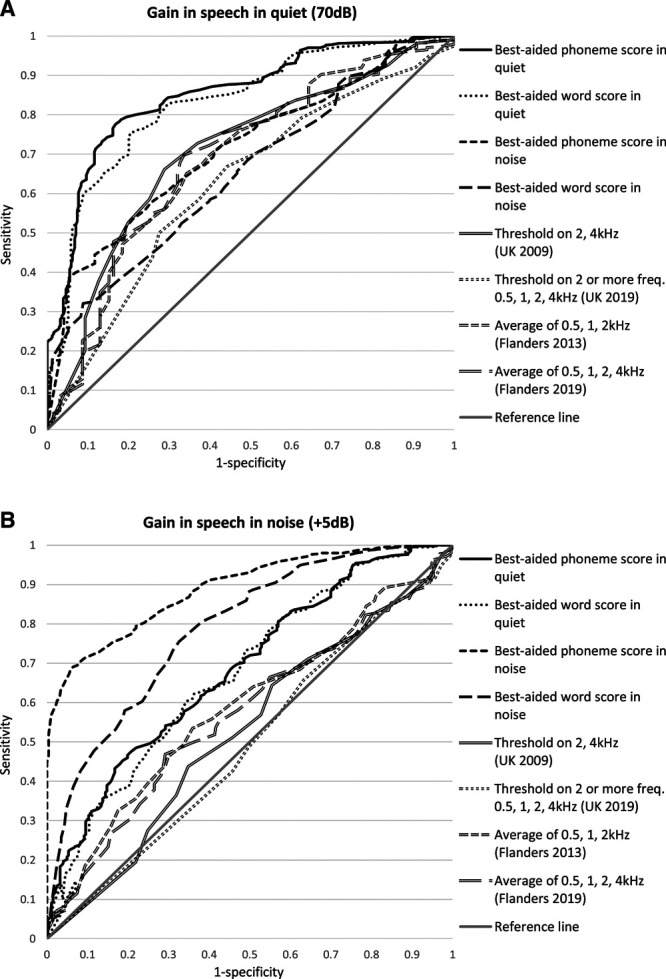
Receiver operator characteristic curves of the preoperative measures used by the Netherlands, United Kingdom, and Flanders. Diagnostic value was analyzed with the binaural outcome of improvement or no improvement (≥0% or <0%) in speech understanding in a quiet setting (A) or in a setting with noise (B) after cochlear implantation (imputed data, n = 552). Thresholds in pure-tone audiometry represent the individual evaluation of each frequency that exceeds a certain cutoff value.

We constructed ROC curves to compare the performance of all preoperative measurements used by the Netherlands, the United Kingdom, and Flanders (Fig. 2). Improved (≥0%) or diminished (<0%) speech understanding after CI was used as a binaural outcome, and the discrimination thresholds of the different preoperative measures were varied to calculate the sensitivity and 1-specificity of each threshold. The best-aided phoneme score in a quiet setting had the highest diagnostic ability for the improvement of speech understanding in a quiet setting, with an area under the curve (AUC) of 0.853, which was significantly higher (*p* < .001) than all other preoperative measures, except best-aided word score in a quiet setting (AUC = 0.830; *p* = .055). Compared with the new British criteria, the old British criteria used a better approach to the PTA for predicting improved speech understanding in a quiet setting, with an AUC of 0.707 for evaluation of degree of HL at 2 and 4 kHz being significantly larger than an AUC of 0.623 for evaluation at two or more frequencies (0.5, 1, 2, 3, and 4 kHz) (*p* = .046). In contrast, the approach to PTA in the old and new Flanders criteria did not differ from each other (AUC of 0.688 for evaluation of the degree of HL at 0.5, 1, and 2 kHz; AUC of 0.687 for evaluation at 0.5, 1, 2, and 4 kHz; *p* = .668).

The best-aided phoneme score in a setting with noise had the highest diagnostic ability for improvement of speech understanding in noise after CI, with an AUC of 0.887, which was significantly higher (*p* < .001) than all other preoperative measures, except best-aided word score in a setting with noise (AUC = 0.784; *p* = .069). The best-aided phoneme and word score in a quiet setting (AUC = 0.684 and 0.678, respectively) had higher diagnostic abilities than the old and new PTA criteria of the United Kingdom (AUC = 0.525 and 0.491, respectively; *p* = .035 and .011) but did not differ from the old and new PTA criteria of Flanders (AUC = 0.586 and 0.567, respectively; *p* = .135 and .064).

## DISCUSSION

In this retrospective study, we evaluated the selection criteria used for adult CI candidacy in the United Kingdom and Flanders. The new criteria introduced in 2019 resulted in a 30% increase of the inclusion of patients with improved speech understanding after CI, as well as sensitivity increase of 31% in both countries. However, the specificity of the new criteria of both countries slightly decreased from 95% to 90%. We found that preoperative best-aided SPT had the highest diagnostic ability for postoperative improvement of speech understanding. This preoperative measurement was dominant for patient selection in the new British and Flemish selection criteria, whereas the PTA prerequisites were more dominant in the old criteria. Notably, the new criteria still resulted in rejection of candidates who would be expected to gain considerably in speech understanding after implantation. Within the excluded groups, only one out of every eight patients did not exhibit postoperative improved speech understanding.

Both Flanders and the United Kingdom recently implemented less restrictive selection criteria for adult CIs (National Institute for Health and Clinical Excellence 2009; De Raeve & Wouters 2013; National Institute for Health and Clinical Excellence 2019). Surprisingly, we found that the old PTA criterion of the United Kingdom was more accurate for selecting CI candidates compared with their new PTA criterion, because higher frequencies (e.g., 2 and 4 kHz) were more accurate for defining which candidates will benefit from a CI than the evaluation of more frequencies (0.5–4 kHz). This finding may be explained by the fact that the patients who lacked postoperative improvement often had residual hearing in the lower frequencies (Francis et al. 2004). However, a previous study revealed that the preoperative best-aided SPT should be used as the selection criterion, because it showed the highest diagnostic ability of all preoperative measures (T. F. K. van der Straaten et al., Reference Note 1). Both the United Kingdom and Flanders selection criteria changed from being dependent on the degree of HL to being more reliant on preoperative SPT. In general, and also in the United Kingdom and Flanders, it would be beneficial to stop using PTA criteria, and to instead use only preoperative best-aided SPT for candidacy selection.

CIs should be provided to candidates who are likely to benefit in terms of speech understanding. Policy-makers frequently discuss the degree of benefit in postoperative speech understanding; however, subjective improvement of speech understanding in daily life varies between patients. While some patients experience a substantial improvement in daily speech understanding with a postoperative score improvement of 5%, other candidates might experience almost no difference in daily listening with a postoperative score improvement of 10%. Moreover, nowadays, candidates with progressive HL are implanted at an earlier stage, while they still have residual hearing (Summerfield & Marshall 1995; Friedland et al. 2003; Gomaa et al. 2003; Cullen et al. 2004; Francis et al. 2004). In this scenario, the anticipated decrease of preoperative speech understanding will be eliminated by the earlier implantation, and the postoperative speech understanding would be stable, albeit sometimes comparable to their preoperative speech understanding while they still had residual hearing. It remains challenging to define selection criteria for these patients of borderline candidacy.

In the present study, we focused only on the CI selection criteria for adults with postlingual HL, not for children with prelingual HL. These groups differ considerably in etiology, age at implantation, and the time during which they could develop language and speech with sufficient auditory input preimplantation (Peterson et al. 2010). In addition, preoperative selection criteria for children are often based on PTA due to the fact that they are not able to complete a preoperative SPT (Lovett et al. 2015). Using PTA criteria in adults resulted in the inclusion of a higher proportion of candidates without postoperative improvement and thus had lower diagnostic ability. It would be interesting to assess the performance of PTA criteria in children with CI. Of course, one should use other measures than in the present study when preoperative speech understanding data are not available.

The relatively lenient criteria and the large number of implantations in the LUMC enabled our present evaluation of the CI selection criteria of the United Kingdom and Flanders. Countries with more lenient CI criteria, such as Germany and Australia, could check the performance of the Dutch selection criteria (Leigh et al. 2016; Vickers et al. 2016; Hoppe et al. 2019). These countries use selection criteria for each individual ear, which enables them to additionally implant patients with asymmetrical or unilateral HL and leads to more bilateral implantations. In the Netherlands, bilateral implantation is not reimbursed for adults. Therefore, the number of adult bilateral CI users in the Netherlands is small, making it hard to analyze the effect on speech understanding. However, the current dataset does allow us to identify the group of users that obtained considerable benefit of implantation in their worst-performing ear relative to their preoperative performance. For example, patients with preoperative best-aided phoneme scores less than 50% who improved this after implantation with more than 20% to 30% in their worst-performing ear. Considering the correlation with preoperative speech understanding, one could expect an even better performance if the best-performing ear was implanted (Hoppe et al. 2019). This would allow to carefully select the patients who will benefit of a second implant, irrespective of the bilateral benefits. Testing the bimodal speech understanding with an additional HA before sequential bilateral implantation would make this selection process even more robust.

The SPT used in our center was identical to the one used in Flanders (Bosman & Smoorenburg 1995) but differed from the one used in the United Kingdom wherefore an estimate of the old sentence and new phoneme criteria was used (Vickers et al. 2013). Notably, the number of candidates who did not exhibit improved speech understanding after implantation in this study may differ from other CI centers depending on multiple factors, such as the surgeon, the amount of preserved residual hearing, type of device, the effort toward rehabilitation, and so on (Peterson et al. 2010).

In conclusion, the criteria newly introduced in Flanders and the United Kingdom resulted in increased sensitivity and increased numbers of patients who will exhibit improved speech understanding after CI. These criteria still resulted in the rejection of candidates who would be successfully implanted in the Netherlands, with only one out of eight of the rejected candidates showing no postoperative improvement. The best-aided SPT had the highest diagnostic ability and would, therefore, be the ideal instrument for CI selection criteria. These findings will improve appropriate selection of CI candidates and help authorities and CI centers to effectively formulate selection criteria for adults with postlingual HL.

## ACKNOWLEDGMENTS

T. F. K. v. d. S. and J. J. B involved in study concept and design. T. F. K. v. d. S. involved in statistical analysis. T. F. K. v. d. S. and J. J. B involved in drafting of the manuscript. All authors involved in critical revision of the manuscript for important intellectual content and acquisition, analysis, or interpretation of data.
